# Impact of Right Ventricular Dilatation in Patients with Atrial Septal Defect

**DOI:** 10.1155/2020/9509105

**Published:** 2020-04-27

**Authors:** Rie Nakayama, Yoichi Takaya, Teiji Akagi, Koji Nakagawa, Nobuhisa Watanabe, Saori Nobusada, Toshi Matsushita, Norihisa Toh, Susumu Kanazawa, Hiroshi Ito

**Affiliations:** ^1^Department of Cardiovascular Medicine, Okayama University Graduate School of Medicine, Dentistry and Pharmaceutical Sciences, Okayama, Japan; ^2^Division of Medical Support, Okayama University Hospital, Okayama, Japan; ^3^Department of Radiology, Okayama University Graduate School of Medicine, Dentistry and Pharmaceutical Sciences, Okayama, Japan

## Abstract

**Objective:**

The aim of this study was to examine the relationship between right ventricular (RV) volume and exercise capacity in adult patients with atrial septal defect (ASD) and to determine the degree of RV dilatation for transcatheter ASD closure.

**Background:**

RV dilatation is an indication of transcatheter ASD closure; however, few studies have reported the clinical significance of RV dilatation.

**Methods:**

We enrolled 82 consecutive patients (mean age, 49 ± 18 years; female, 68%) who underwent cardiac magnetic resonance imaging and symptom-limited cardiopulmonary exercise test before ASD closure. The relationship between RV volume and peak oxygen uptake (VO_2_) was evaluated.

**Results:**

The mean RV end-diastolic volume index was 108 ± 27 ml/m^2^ (range, 46 to 180 ml/m^2^). The mean peak VO_2_ was 24 ± 7 ml/min/kg (range, 14 to 48 ml/min/kg), and the mean predicted peak VO_2_ was 90 ± 23%. There were significant negative relationships of RV end-diastolic volume index with peak VO_2_ (*r* = −0.28, *p* < 0.01) and predicted peak VO_2_ (*r* = −0.29, *p* < 0.01). The cutoff value of RV end-diastolic volume index <80% of predicted peak VO_2_ was 120 ml/m^2^, with the sensitivity of 49% and the specificity of 89%.

**Conclusions:**

There was a relationship between RV dilatation and exercise capacity in adult patients with ASD. RV end-diastolic volume index ≥120 ml/m^2^ was related to the reduction in peak VO_2_. This criterion of RV dilatation may be valuable for the indication of transcatheter ASD closure.

## 1. Introduction

Atrial septal defect (ASD) is the most common congenital heart disease with a left-to-right shunt in adults and is recognized in 7% of all congenital heart diseases [1]. Many patients with ASD are asymptomatic in adulthood, but a reduction in exercise capacity and exertional shortness of breath appear at over 40 years old [2–5]. Currently, transcatheter closure is a general therapy for secundum ASD [6, 7]. A significant left-to-right shunt causing right ventricular (RV) dilatation is an accepted indication of ASD closure [8]. Several studies have reported that transcatheter ASD closure results in RV remodeling and exercise capacity improvement [9–13]. However, the relationship between RV dilatation and the reduction in exercise capacity before ASD closure remains unclear. Furthermore, there is no criterion of RV dilatation for performing ASD closure. Cardiac magnetic resonance (CMR) imaging can accurately quantify RV volume. Thus, we hypothesized that RV dilatation evaluated by CMR imaging was closely related to exercise capacity. The aim of this study was to examine the relationship between RV volume and exercise capacity in patients with ASD and to determine the degree of RV dilatation for ASD closure.

## 2. Methods

### 2.1. Study Population

We enrolled 82 consecutive adult patients with secundum ASD who underwent CMR imaging and symptom-limited cardiopulmonary exercise test (CPET) from January 2016 to August 2019 at Okayama University Hospital. Patient population in this study was 36% of all patients who underwent ASD closure. Indications for ASD closure were a significant left-to-right shunt, RV volume overload, and/or clinical symptoms of heart failure or paradoxical embolism. Patients with severe pulmonary arterial hypertension who received specific medications were excluded. After right heart catheterization was performed for hemodynamic evaluation such as pulmonary to systemic flow ratio (Qp/Qs), mean pulmonary artery pressure, cardiac output, and cardiac index, transcatheter ASD closure was performed in all patients without any complications using an Amplatzer septal occluder device (Abbott, Chicago, IL, USA) or an Occlutech Figulla Flex II device (Occlutech GmbH, Jena, Germany).

All patients gave written informed consent for the examinations. The study was approved by the ethical committee of our institution.

### 2.2. Cardiac Magnetic Resonance Imaging

CMR imaging studies were performed on a 1.5-T scanner (Siemens AG, Erlangen, Germany) under electrocardiogram synchronization before ASD closure. The endocardial and epicardial contours of both ventricles were manually traced on the short axis end-diastolic and end-systolic phases using software (Ziostation; Ziosoft, Tokyo, Japan). RV end-diastolic volume, RV end-systolic volume, RV ejection fraction, left ventricular (LV) end-diastolic volume, and LV end-systolic volume were evaluated.

### 2.3. Echocardiography

Transthoracic echocardiography was performed in all patients before ASD closure. RV end-diastolic basal diameter, RV end-diastolic area, and RV end-systolic area were evaluated in the apical 4-chamber view. To assess RV function, tricuspid annular plane systolic excursion (TAPSE), tricuspid annular systolic velocity (S), and RV fractional area change (FAC) were measured. LV end-diastolic diameter, LV end-systolic diameter, and LV ejection fraction were also measured. ASD diameter was measured by transesophageal echocardiography.

### 2.4. Cardiopulmonary Exercise Test

A maximum symptom-limited CPET was performed using a bicycle ergometer before ASD closure. The workload was increased by 15 watts/min. Patients exercised until exhaustion. Oxygen uptake (VO_2_), carbon dioxide production (VCO_2_), and minute ventilation (VE) were assessed by breath-by-breath gas analysis. Peak VO_2_, predicted peak VO_2_, peak work, ventilatory equivalent for carbon dioxide (VE/VCO_2_ slope), and respiratory exchange ratio were evaluated. Peak VO_2_ was defined as the highest value recorded during the last minute of exercise. Predicted peak VO_2_ was determined by age and sex.

### 2.5. Statistical Analysis

Data are presented as mean ± standard deviation for continuous variables and as number and percentage for categorical variables. Pearson's correlation coefficient was used to assess relationships between CMR imaging and echocardiographic parameters with exercise capacity. Linear regression analysis was performed for multivariate analysis. The cutoff value of RV volume for the reduction in exercise capacity was estimated using receiver operating characteristic curve. Statistical analysis was performed with JMP version 14.0 (SAS Institute Inc., Cary, NC, USA), and significance was defined as a value of *p* < 0.05.

## 3. Results

### 3.1. Patient Characteristics

The study population consisted of 82 patients (female: 68%) with mean age of 49 ± 18 years. Patient characteristics are shown in Table 1. The majority of patients had New York Heart Association functional class I. ASD diameter was 15 ± 6 mm. The Qp/Qs was 2.2 ± 0.8, and the mean pulmonary artery pressure was 16 ± 5 mmHg.

### 3.2. Cardiac Magnetic Resonance Imaging and Echocardiography

CMR imaging and echocardiographic parameters are summarized in Table 2. CMR imaging showed RV end-diastolic volume index of 108 ± 27 ml/m^2^ (range, 46 to 180 ml/m^2^) and RV end-systolic volume index of 78 ± 19 ml/m^2^ (range, 34 to 122 ml/m^2^). Echocardiography showed RV end-diastolic basal diameter of 46 ± 6 mm, RV end-diastolic area of 27 ± 6 cm^2^, and RV end-systolic area of 15 ± 3 cm^2^. As the parameters of RV function, TAPSE was 25 ± 4 mm, S was 15 ± 2 cm/s, and RV FAC was 45 ± 4%.

### 3.3. Cardiopulmonary Exercise Test

CPET parameters are summarized in Table 2. Peak VO_2_ was 24 ± 7 ml/min/kg (range, 14 to 48 ml/min/kg), and predicted peak VO_2_ was 90 ± 23%. Fifty-one patients (62%) had predicted peak VO_2_ ≥ 80%, 30 patients (37%) had predicted peak VO_2_ 60–80%, and one patient (1%) had predicted peak VO_2_ <60%.

### 3.4. Relationships of Cardiac Magnetic Resonance Imaging and Echocardiography with Cardiopulmonary Exercise Test

**Figure 1:**
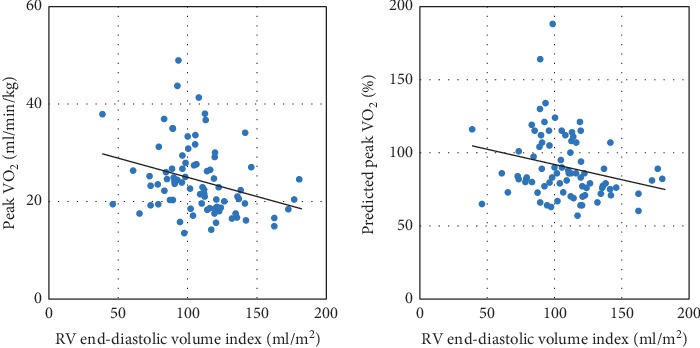
Relationships of RV volume with exercise capacity. There were significant negative relationships of RV end-diastolic volume index with peak VO_2_ and predicted peak VO_2_. RV = right ventricular; VO_2_ = oxygen uptake.

Regarding CMR imaging, the enlargement of RV end-diastolic volume index was related to the reduction in peak VO_2_ (*r* = −0.29, *p* < 0.01) and predicted peak VO_2_ (*r* = −0.24, *p*=0.03) (Figure 1). RV ejection fraction was negatively related to peak VO_2_ (*r* = −0.30, *p* < 0.01). There were no relationships of LV end-diastolic volume index or LV end-systolic volume index with exercise capacity (Table 3). Multivariate linear regression analysis showed that RV end-diastolic volume index was independently related to the reduction in peak VO_2_ (Table 4).

Regarding echocardiography, RV end-diastolic basal diameter and RV end-diastolic area were related to the reduction in peak VO_2_ (*r* = −0.30, *p* < 0.01; *r* = −0.25, *p*=0.03, respectively). TAPSE, S, or RV FAC was not related to exercise capacity.

### 3.5. Degree of Right Ventricular Dilatation

The optimal cutoff value of RV end-diastolic volume index <80% of predicted peak VO_2_ was 120 ml/m^2^ (area under the curve = 0.69), with the sensitivity of 49% and the specificity of 89%.

## 4. Discussion

The major findings of the present study were as follows: (1) there was a significant negative relationship between RV end-diastolic volume and exercise capacity in patients with ASD; (2) RV end-diastolic volume was the independent factor related to reduced peak VO_2_; and (3) the cutoff value of RV end-diastolic volume index for reduced peak VO_2_ was 120 ml/m^2^. To the best of our knowledge, this study is the first study to show the relationship between RV dilatation and the reduction in exercise capacity before ASD closure and to indicate the criterion of RV dilatation for reduced peak VO_2_.

Most patients with ASD have impaired exercise capacity with age [14, 15]. RV volume overload because of a left-to-right shunt causes enlargement of the right ventricle, leading to clinical symptoms such as dyspnea and fatigue. Exercise capacity depends on the left-to-right shunt and RV volume overload. RV dilatation may represent a limiting factor. However, the relationship between RV dilatation and peak VO_2_ before ASD closure has not been fully investigated.

Reduced exercise capacity is associated with decreased LV stroke volume and cardiac output in patients with ASD [12]. Altered interventricular interaction induced by long-standing RV volume overload plays a central role in limiting exercise tolerance [14]. Because of RV dilatation, the interventricular septum bulges paradoxically and encroaches into LV cavity, impairing LV filling and thus both diastolic and systolic performance [16–18]. This mechanism could cause decreased cardiac output, leading to reduced peak VO_2_. After transcatheter ASD closure, the abolishment of the left-to-right shunt leads to augmented LV filling by preload and therefore to increased cardiac output [14]. Many studies have reported that transcatheter ASD closure results in a positive RV remodeling and improved exercise capacity [9–13]. The improvement in cardiac form following increased cardiac output is the most likely factor leading to improved peak VO_2_ [19]. In this study, cardiac output was not markedly decreased. It might be compensated by RV hypercontraction with the dilatation. However, RV end-diastolic volume tended to be negatively related to cardiac output. Further studies are needed in order to investigate the relationship between RV dilatation and decreased cardiac output.

In the present study, RV end-diastolic volume was evaluated by CMR imaging. CMR imaging can be used to accurately quantify RV volume, although there are certain limitations in echocardiography for assessing RV volume because of the complex anatomy and structure of the right ventricle. Even three-dimensional echocardiography is inadequate [20]. These factors may influence our result that RV dilatation evaluated by CMR imaging was closely related to exercise capacity.

The present study showed that RV ejection fraction was negatively related to peak VO_2_. Following Starling's law of the heart, the increased preload results in high ventricular ejection function. The parameters of RV function such as RV ejection fraction, TAPSE, S, and RV FAC tend to be elevated in patients with ASD and are decreased after device closure [21–23]. The degree of RV ejection fraction depends on RV volume overload. Therefore, the relationship between RV ejection fraction and peak VO_2_ may be induced by RV volume overload, similar to the relationship with RV dilatation. With regards to echocardiographic parameters, TAPSE, S, or RV FAC was not related to exercise capacity. The long axis RV function alone such as TAPSE and S and the radial RV function alone such as RV FAC may be insufficient for the functional assessments.

### 4.1. Clinical Implications

A significant left-to-right shunt with RV dilatation or Qp/Qs > 1.5 is indication of transcatheter ASD closure [8]. The value of Qp/Qs is widely used for the planning of therapeutic strategies. However, the clinical significance of RV dilatation, including the relationship with clinical symptoms, has not been well investigated. Further, the degree of RV dilatation that requires transcatheter ASD closure remains unclear. The present study showed that RV dilatation was related to exercise capacity in asymptomatic or mildly symptomatic patients. RV end-diastolic volume ≥120 ml/m^2^ predicted the reduction of peak VO_2_. Our results support that RV dilatation is valuable for determining the optimal timing of transcatheter ASD closure.

### 4.2. Study Limitations

There are some limitations in the present study. First, the number of patients was small. Large studies are required to confirm our findings. Second, exercise capacity was influenced by several factors, but noncardiac factors such as pulmonary function and skeletal muscle function were not evaluated. Third, some patients with atrial fibrillation were included in this study. The presence of atrial fibrillation might affect exercise capacity. Finally, the RV ejection fraction value was low compared with previous studies [24]. Thus, the calculation of RV ejection fraction might be underestimated in this study.

## 5. Conclusions

There was a relationship between RV dilatation and exercise capacity in adult patients with ASD. RV end-diastolic volume index ≥120 ml/m^2^ was related to the reduction in peak VO_2_. Our findings suggest that this criterion of RV dilatation is valuable for the indication of transcatheter ASD closure.

## Figures and Tables

**Table 1:** Patient characteristics.

Variables	
Age (yrs)	49 ± 18
Female	56 (68%)
Body surface area (m^2^)	1.6 ± 0.2
New York Heart Association functional class I	63 (%)
New York Heart Association functional class II	35 (%)
New York Heart Association functional class III	1 (%)
Atrial fibrillation	5 (6%)
Diuretics	11 (13%)
ASD diameter (mm)	15 ± 6
Qp/Qs	2.2 ± 0.8
Mean pulmonary artery pressure (mmHg)	16 ± 5
Cardiac output, (L/min)	4.3 ± 1.3
Cardiac index, (L/min/m^2^)	2.7 ± 0.8

Data are presented as mean ± standard deviation or number (%) of patients. ASD = atrial septal defect; Qp/Qs = pulmonary to systemic flow ratio.

**Table 2:** CMR imaging, echocardiography, and CPET parameters.

Variables	
*CMR imaging*	
RV end-diastolic volume index (ml/m^2^)	108 ± 27
RV end-systolic volume index (ml/m^2^)	78 ± 19
RV ejection fraction (%)	27 ± 7
LV end-diastolic volume index (ml/m^2^)	54 ± 12
LV end-systolic volume index (ml/m^2^)	30 ± 9
*Echocardiography*	
RV end-diastolic basal diameter (mm)	46 ± 6
RV end-diastolic area (cm^2^)	27 ± 6
RV end-systolic area (cm^2^)	15 ± 3
RV FAC (%)	45 ± 4
TAPSE (mm)	25 ± 4
S (cm/s)	15 ± 2
LV end-diastolic diameter (mm)	41 ± 4
LV end-systolic diameter (mm)	26 ± 4
LV ejection fraction (%)	66 ± 9
*CPTE*	
Peak oxygen uptake (ml/kg/min)	24 ± 7
Predicted peak oxygen uptake (%)	90 ± 23
Peak work (watts)	104 ± 36
Ventilatory equivalent for carbon dioxide slope	23 ± 3
Respiratory exchange ratio	1.2 ± 0.1

Data are presented as mean ± standard deviation. CMR = cardiac magnetic resonance; CPET = cardiopulmonary exercise test; FAC = fractional area change; LV = left ventricular; RV = right ventricular; S = tricuspid annular systolic velocity; TAPSE = tricuspid annular plane systolic excursion.

**Table 3:** Relationships of CMR imaging and echocardiography with exercise capacity.

Variables	Peak VO_2_		Predicted peak VO_2_	
	*r*	*p* value	*r*	*p* value
*CMR imaging*				
RV end-diastolic volume index	−0.29	<0.01	−0.24	0.03
RV end-systolic volume index	0.05	0.15	0.13	0.23
RV ejection fraction	−0.30	<0.01	−0.24	0.03
LV end-diastolic volume index	0.04	0.09	0.03	0.78
LV end-systolic volume index	0.11	0.34	0.03	0.78
*Echocardiography*				
RV basal diameter	−0.30	<0.01	−0.09	0.43
RV end-diastolic area	−0.25	0.03	−0.09	0.41
RV end-systolic area	−0.24	0.03	−0.08	0.47
RV FAC	0.05	0.69	0.02	0.83
TAPSE	0.05	0.66	0.09	0.83
S	0.14	0.21	<0.01	0.94
LV end-diastolic diameter	0.22	0.04	0.02	0.85
LV end-systolic diameter	0.22	0.04	<0.01	0.97
LV ejection fraction	0.08	0.45	0.04	0.27

CMR = cardiac magnetic resonance; FAC = fractional area change; LV = left ventricular; RV = right ventricular; S = tricuspid annular systolic velocity; TAPSE = tricuspid annular plane systolic excursion; VO_2_ = oxygen uptake.

**Table 4:** Multivariate linear regression analysis for peak VO_2_.

Variables	Beta	95% of CI	*p* value
RV end-diastolic volume index	−0.35	(−0.10, −0.008)	0.02
Mean pulmonary artery pressure	0.01	(−031, 0.34)	0.91
Cardiac output	0.15	(−0.75, 2.33)	0.31
Qp/Qs	0.20	(−1.05, 4.28)	0.23
ASD diameter	−0.05	(−0.37, 0.26)	0.74
RV FAC	0.08	(−0.29, 0.60)	0.51
TAPSE	−0.05	(−0.62, 0.45)	0.76
S	0.20	(−0.30, 0.60)	0.16

ASD = atrial septal defect; CI = confidence interval; FAC = fractional area change; Qp/Qs = pulmonary to systemic flow ratio; RV = right ventricular; S = tricuspid annular systolic velocity; TAPSE = tricuspid annular plane systolic excursion; VO_2_ = oxygen uptake.

## Data Availability

The data used to support the findings of this study are available from the corresponding author upon request.

## Abbreviations and Acronyms

ASD: Atrial septal defect
CMR: Cardiac magnetic resonance
CPET: Cardiopulmonary exercise test
FAC: Fractional area change
LV: Left ventricular
Qp/Qs: Pulmonary to systemic flow ratio
RV: Right ventricular
S: Tricuspid annular systolic velocity
TAPSE: Tricuspid annular plane systolic excursion
VCO_2_: Carbon dioxide production
VE: Minute ventilation
VO_2_: Oxygen uptake.
